# Editorial: Interactions between NAFLD and cardiac conduction, structure and function: recent advances and treatments

**DOI:** 10.3389/fendo.2023.1334227

**Published:** 2023-11-22

**Authors:** Christiano Argano

**Affiliations:** Department of Internal Medicine, National Relevance and High Specialization Hospital Trust ARNAS Civico, Di Cristina, Benfratelli, Palermo, Italy

**Keywords:** NAFLD, MAFLD, atrial fibrillation, atrial remodeling, left ventricular diastolic dysfunction, monocyte-to-high-density lipoprotein ratio (MHR), heart failure, mortality

Non-alcoholic fatty liver disease (NAFLD) represents the most common liver disease globally (1), and its prevalence has been rising along with the obesity and type 2 diabetes epidemic (2). According to a recent systematic review, the global prevalence of NAFLD increased from 25.3% in 1990-2006 to 38.0% in 2016-2019 (3). Consequently, NAFLD is becoming a major public healthcare challenge (4). It causes a deterioration of quality of life, which increases with disease progression and is worsened by comorbidities (5, 6), representing the leading cause of liver-related morbidity and mortality (7). NAFLD does not only affect the liver but was shown to be a multisystem disease with increased risk of type 2 diabetes, dyslipidaemia, metabolic syndrome, hypertension, kidney and cardiovascular diseases (8). Increasing literature data suggests that subjects with NAFLD show myocardial functional and structural changes, leading to cardiac remodelling and increased risk of heart failure (HF) (9). In particular, different studies showed associations between NAFLD and left ventricular and diastolic dysfunction (9, 10), right ventricular dysfunction (11) and left ventricular hypertrophy (12). Moreover, recent data showed that NAFLD is associated with an increased risk of incident atrial fibrillation (AF) (13) and subjects with higher hepatic fibrosis index were associated with increased AF risk (14).

Mechanisms that link NAFLD to cardiac functional and structural changes, apart from the common risk factors, have yet to be explored. In NAFLD, insulin resistance, inflammation and oxidative stress may lead to cardiac insulin resistance and cardiac fibrosis, determining an alteration in cardiac structure (15). Moreover, the increased myocardial fatty acid oxidation, which is less efficient than glucose metabolism, may contribute to the development of heart failure (16).

This Research Topic aimed to provide insight into several aspects of the connections between NAFLD cardiac conduction, structure and function, presenting novelties on the potential pathological mechanism at the basis of these relationships. Overall, four original articles and two meta-analyses have been published.

In this Research Topic, Peng et al. after underlining the importance of the current debate on renaming NAFLD to metabolic dysfunction associated fatty liver disease (MAFLD), aimed to examine whether MAFLD is associated with left ventricular diastolic dysfunction and cardiac remodelling. Additionally, the authors try to identify the impact of different subgroups (lean, overweight/obese and diabetes) and the severity of MAFLD (normal, mild, moderate, and severe hepatic steatosis). Left ventricular diastolic dysfunction was significantly more prevalent in the MAFLD group than in the normal group. The overweight and diabetes subgroups were significantly associated with cardiac alterations such as interventricular septum thickness, LV posterior wall thickness, left atrial diameter, relative wall thickness, and LV mass index. In addition, subjects with moderate to severe steatosis had higher risks for left ventricular diastolic dysfunction and cardiac remodelling.

Decoin et al. brought new insights into the association between NAFLD/MAFLD and atrial fibrillation. The authors showed that the presence of MAFLD patients at risk of liver fibrosis is associated with adverse atrial remodelling, particularly an increase in LA volume, impaired LA reservoir function, and increased low-voltage areas. In addition, the liver fibrosis scoring in MAFLD patients predicted AF recurrence after ablation. In MAFLD subjects with AF recurrence, high liver fibrosis scores presented a higher AF burden. The most significant conclusion to be drawn is that liver fibrosis scoring in MAFLD patients is associated with adverse atrial remodelling and AF recurrences following catheter ablation.

Wang et al. proposed a novel biomarker of inflammation and oxidative stress, such as Monocyte-to- high-density lipoprotein ratio (MHR), as a predictor of the risk of AF among NAFLD patients. A retrospective cross-sectional analysis in a single-center sample was performed. MHR was significantly higher in patients with NAFLD with AF in comparison with NAFLD subjects without AF. MHR could be a simple and practical new inflammatory index used to assess the risk of AF in the clinical management of NAFLD patients.

Zhou et al. performed an updated systematic review and meta-analysis of cohort studies to define better the correlation between NAFLD/MAFLD and the likelihood of developing AF. Current updated evidence showed that NAFLD may be linked to a slightly higher risk of developing AF, particularly among Asian populations and those diagnosed with NAFLD using fatty liver index criteria. Authors highlighted the concept that further studies should consider factors such as specific population, the severity of NAFLD/MAFLD, diagnostic methods of NAFLD and AF, and cardiometabolic risk factors to determine better the association between NAFLD and the risk of AF.

Qiu et al. performed a meta-analysis to explore the association between NAFLD and the risk of adverse outcomes in patients with HF. A total of six studies involving 12,374 patients with HF were included for analysis, with a median follow-up duration of 2.5 years. The pooled analysis showed that, after adjusting for multiple cardiovascular risk factors, HF patients with NAFLD were associated with a significantly increased risk of major adverse outcomes, all-cause mortality and HF hospitalization or re-hospitalization.

Finally, Jiang et al. investigated the associations of NAFLD and its advanced fibrosis with heart failure with preserved ejection fraction (HFpEF) according to obesity, glycated haemoglobin A1c (HbA1c), blood pressure (BP), and low-density lipoprotein cholesterol (LDL-C) goal achievement status in 2,418 hospitalized patients with type 2 diabetes. This cross-sectional analysis showed that in patients with type 2 diabetes, simple steatosis was not associated with HFpEF risk compared with patients without steatosis, and advanced hepatic fibrosis was significantly associated with an increased risk of HFpEF, regardless of obesity status, HbA1c, BP, and LDL-C goal achievement status.

Taken together, the studies published in this Research Topic provide the reader with an increased understanding of interactions between NAFLD and cardiac conduction, structure and function, opening new scenarios in the evaluation and treatment of subjects suffering from NAFLD. Further studies are needed to translate these observations into the real-world management of NAFLD patients.

## Author contributions

CA: Writing – original draft, Conceptualization.
